# Interpenetrating interfaces for efficient perovskite solar cells with high operational stability and mechanical robustness

**DOI:** 10.1038/s41467-021-21292-3

**Published:** 2021-02-12

**Authors:** Qingshun Dong, Chao Zhu, Min Chen, Chen Jiang, Jingya Guo, Yulin Feng, Zhenghong Dai, Srinivas K. Yadavalli, Mingyu Hu, Xun Cao, Yuqian Li, Yizhong Huang, Zheng Liu, Yantao Shi, Liduo Wang, Nitin P. Padture, Yuanyuan Zhou

**Affiliations:** 1 State Key Laboratory of Fine Chemicals, Department of Chemistry, School of Chemical Engineering, Dalian University of Technology, Dalian, 116024 China; 2 School of Engineering, Brown University, Providence, 02912 RI USA; 3 SEU-FEI Nano-Pico Center, Key Laboratory of MEMS of Ministry of Education, Collaborative Innovation Center for Micro/Nano Fabrication, Device and System, Southeast University, Nanjing, 210096 China; 4 School of Materials Science and Engineering, Nanyang Technological University, Nanyang Avenue, 639798 Singapore; 5 Analysis and Test Center, Beijing University of Chemical Technology, Beijing, 100029 China; 6 Department of Chemistry, Tsinghua University, Beijing, 100084 China; 7 Department of Physics, Hong Kong Baptist University, Kowloon, Hong Kong SAR, China

**Keywords:** Chemistry, Energy science and technology, Materials science, Physics

## Abstract

The perovskite solar cell has emerged rapidly in the field of photovoltaics as it combines the merits of low cost, high efficiency, and excellent mechanical flexibility for versatile applications. However, there are significant concerns regarding its operational stability and mechanical robustness. Most of the previously reported approaches to address these concerns entail separate engineering of perovskite and charge-transporting layers. Herein we present a holistic design of perovskite and charge-transporting layers by synthesizing an interpenetrating perovskite/electron-transporting-layer interface. This interface is reaction-formed between a tin dioxide layer containing excess organic halide and a perovskite layer containing excess lead halide. Perovskite solar cells with such interfaces deliver efficiencies up to 22.2% and 20.1% for rigid and flexible versions, respectively. Long-term (1000 h) operational stability is demonstrated and the flexible devices show high endurance against mechanical-bending (2500 cycles) fatigue. Mechanistic insights into the relationship between the interpenetrating interface structure and performance enhancement are provided based on comprehensive, advanced, microscopic characterizations. This study highlights interface integrity as an important factor for designing efficient, operationally-stable, and mechanically-robust solar cells.

## Abstract (web summary)

Operational stability and mechanical robustness remain as engineering bottlenecks in perovskite solar cells technology. Here, Dong et al. introduce an interpenetrating perovskite at the electron-transporting-layer interface that enables a 1000-hour stable operation and high endurance against bending fatigue over 2500 cycles.

## Introduction

The past decade has witnessed the emergence of perovskite solar cells (PSCs) as a disruptive photovoltaic (PV) technology^1–4^. The state-of-the-art PSCs generally use organic–inorganic halide perovskites (OIHPs) with compositions based on formamidinium lead iodide (FAPbI_3_) or methylammonium lead iodide (MAPbI_3_), and they can deliver power conversion efficiencies (PCEs) up to 25.5%, rivalling polycrystalline silicon solar cells^5^. The high PCEs are attributed to favorable optoelectronic properties of OIHPs, including high absorption coefficients, long carrier diffusion lengths, and high defect tolerance^6–8^. Also, OIHPs can be solution-processed at low temperatures, which enables the fabrication of lightweight and flexible PSCs^9–12^. Such combination of high performance and variable functionality makes PSCs appealing to numerous practical applications such as building-integrated PVs. Despite this promise, the deployment of PSCs has been held back due to concerns regarding their stability^13^. In this regard, there is a significant ongoing effort to make PSCs more stable while not compromising the device PCE.

Interface engineering is one of the most promising approaches for making efficient stable PSCs^14–16^. Conventional interfacial engineering entails either insertion of additional device layers (inorganic nanoparticles, polymers, molecules, etc.) or modification of surfaces using functional organic groups (thiophene, pyridine, etc.) and inorganic dopants (chlorine, alkali, etc.)^17–19^. This is expected to improve the PCE and stability via optimizing energy-level alignment, improving interface contacts, suppressing structural defects, mitigating photocurrent hysteresis, or tailoring surface hydrophobicity^14,17,20^. But these interface-engineering methods may involve additional processing steps, possibly compromise the mechanical integrity of the interfaces in the resulting devices. Thus, it is critical to acquire device interfaces that are both functionally beneficial and mechanically robust for stable long-term device operation under continuous illumination as well as high endurance against repeated cyclic-bending (for flexible devices).

Herein we present a holistic design of an interpenetrating interface which is synthesized by deliberately reacting a pre-deposited FAI-incorporated SnO_2_ (FI–SnO_2_) ETL layer and a PbI_2_-excess OIHP layer. Note that the SnO_2_–OIHP interface^21–24^ is chosen for a proof-of-concept demonstration for the new interface design. This fabrication approach is simple and potentially amenable to scalable processes compared with those similar interface structures previously reported^25–27^. Advanced characterizations, including time-of-flight secondary ion mass spectrometry (TOF–SIMS) and transmission electron microscopy (TEM), have been employed to confirm the interpenetrating structure. By probing the potential profile across the OIHP/ETL interpenetrating interface using Kelvin probe force microscopy (KPFM), we observed positive effects of the FI–SnO_2_ ETL on the heterojunction, electric field, and carrier dynamics. The champion PSC (rigid version) with this interface shows a high PCE of 22.2%. After 1000 h continuous operation under one-sun intensity illumination, this device retains 82% of the initial PCE. We also demonstrate the use of this interface in flexible PSCs, which results in PCEs up to 20.1%. The devices show remarkable mechanical endurance to repeated cyclic-bending fatigue, with a PCE retention of 85% after 2500 cycles, which is related to the enhanced structural integrity of this new interface as revealed by ex-situ cross-sectional scanning electron microscopy (SEM) characterization.

## Results

### Synthesis and characterization of the interpenetrating SnO_2_–OIHP interface

**Fig. 1:**
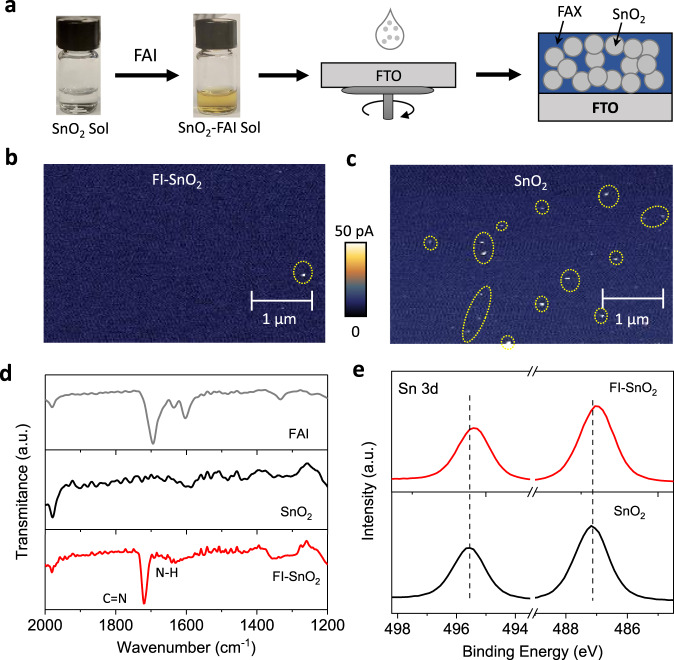
ETL preparation and characterization. **a** Schematic illustration showing the synthesis process of FI–SnO_2_ ETL. **b**, **c** C-AFM mapping of the FI–SnO_2_ (left) and pristine SnO_2_ (right) ETLs on FTO substrates. **d** FTIR spectra of FAI, SnO_2_, and FI–SnO_2_. **e** XPS spectra for Sn 3*d* of FI–SnO_2_ and pristine SnO_2_ ETLs.

Figure 1a illustrates the preparation process for the FI–SnO_2_ ETL. A certain amount of FAI powder was first dissolved in an as-prepared SnO_2_-nanocrystals colloidal solution in isopropanol (IPA), which turns the solution from colorless to brownish color. Meanwhile, the FAI in the colloid has triggered the re-growth of SnO_2_ nanocrystals, probably caused by the hydrolysis reaction initiated by the OH^−^ generated during the oxidation of I^−^ (see Supplementary Fig. 1)^28^. Then, the solution was spin-coated on an FTO-coated glass substrate, followed by thermal annealing at 80 °C for 2 h in the air and a sequential UV–ozone (UVO) treatment for 10 min. The concentration of FAI in the SnO_2_ colloidal solution was optimized to 10 mg mL^−1^ based on the photovoltaic performance of the resulting PSCs (see Supplementary Fig. 2). The surface morphology of FI–SnO_2_ ETL was examined using scanning electron microscopy (SEM) and atomic force microscopy (AFM), and it appears to be very uniform, similar to that of pristine SnO_2_ ETL (see Supplementary Fig. 3). We further obtained conducting-AFM (C-AFM) maps for both FI–SnO_2_ and pristine SnO_2_ ETLs as shown in Fig. 1b and c, respectively. While the electrical conductivity is uniform for both samples, more shunting locations are seen in the pristine SnO_2_ ETL, which can be attributed to the existence of more pinholes that allow direct contacts between the FTO and C-AFM tip^28^. This indicates that in the presence of FAI, SnO_2_ nanocrystals are more uniformly distributed within the layer. We analyzed the composition of the FI–SnO_2_ ETL. Figure 1d shows the Fourier-transform infrared spectroscopy (FTIR) spectrum of FI–SnO_2_ together with the spectra from pristine FAI and SnO_2_ films. Two characteristic transmission bands at the wavenumbers of 1720 cm^−1^ and 1640 cm^−1^ are observed for FI–SnO_2_, which are assigned to the vibrations of the C = N and the N–H bonds, respectively, associated with FA^+^ ions^29^. This suggests the presence of FA^+^ ions in the ETL despite the thermal annealing and UVO treatments during the ETL preparation. X-ray photoelectron spectroscopy (XPS) spectra were also obtained for FI–SnO_2_ and pristine SnO_2_ films (Supplementary Figure 4). Besides N 1 *s* peak (at 400 eV binding energy) for N–H, I 3*d* peak (at 620 eV binding energy) and Cl 2*p* peak (at 199 eV binding energy) are both very evident for FI–SnO_2_, indicating the FAI has been partially converted to FACl. Importantly, as shown in Fig. 1e, the Sn 3*d* doublet peaks for FI–SnO_2_ shift to lower binding energies as compared to those for pristine SnO_2_. This is mostly attributed to the chemical interaction of Sn with less electronegative I and Cl in FA halide. All these results confirm that FI–SnO_2_ exhibits a nanocomposite thin film structure with SnO_2_ nanoparticles uniformly dispersed within an FA mixed halide (FAI and FACl) matrix, as schematically illustrated in the right panel of Fig. 1a.

**Fig. 2:**
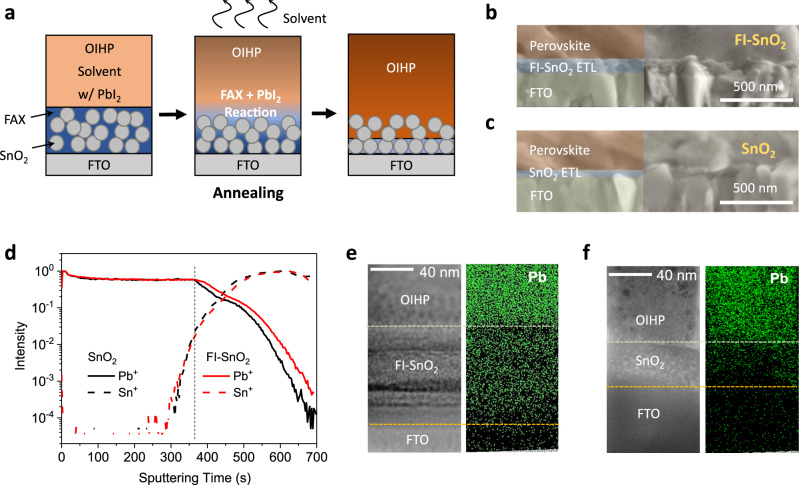
Characterization of SnO_2_-OIHP interfaces. **a** Schematic illustration showing the formation process of the OIHP/FI–SnO_2_ interface by the reaction between the excess PbI_2_ in the OIHP layer and the FA halide in the FI–SnO_2_ ETL. Cross-sectional SEM images of OIHP thin films on, **b** FI–SnO_2_ ETL and, **c** pristine SnO_2_ ETL. **d** TOF–SIMS depth elemental profiles of Pb and Sn into the OIHP/FI–SnO_2_ and the OIHP/SnO_2_ films. Cross-sectional STEM image and EDX Pb-element mapping of OIHP films on, **e** FI–SnO_2_ ETL and, **f** pristine SnO_2_ ETL.

Once the FI–SnO_2_ ETL is prepared, we deposited the OIHP layer using a reported method^30^. Here, we used a precursor solution containing multi-ion compositions of PbI_2_, PbBr_2_, CsI, FAI, and MABr (molar ratio: 1.15: 0.2: 0.05: 1.05: 0.2) in a mixed solvent of dimethylformamide (DMF) and dimethylsulfoxide (DMSO) (volume ratio: 4:1). The resulting OIHP thin film has a Cs_0.04_(FA_0.84_MA_0.16_)_0.96_Pb(I_0.84_Br_0.16_)_3_ composition with a slight excess of PbI_2_. The OIHP thin film was then annealed at 100 °C for 50 min. During annealing, as shown schematically in Fig. 2a, the excess PbI_2_ in the OIHP thin film is expected to react with the FA halide in the FI–SnO_2_ ETL, resulting in a partial infiltration of the OIHP phase into FI–SnO_2_ ETL. Figure 2b and c show cross-sectional SEM images of the as-deposited OIHP thin films on FI–SnO_2_ and pristine SnO_2_ ETLs, respectively. Both OIHP thin films exhibit dense polycrystalline microstructures. We used time-of-flight secondary ion mass spectrometry (TOF–SIMS) to probe the through-thickness elemental distributions of Pb and Sn in the films. In order to exclude the influence of FTO (which contains Sn), OIHP/ETL films for TOF–SIMS analyses were deposited on bare glass substrates. Figure 2d shows the depth profiles of Pb and Sn elemental concentrations revealed by TOF–SIMS (see the depth profiles of other elements in Supplementary Figure 5). With an increase in the sputtering time (corresponding to the depth into the film), for both films, the concentration of Pb (from OIHP) decreases whereas the Sn concentrations (from SnO_2_) increase. The vertical dashed line in Fig. 2d marks the beginning of the OIHP/ETL interface. As compared to the sample with the pristine SnO_2_ ETL, the Pb profile for the sample with the FI–SnO_2_ ETL exhibits a relatively shallower slope at the interface, which provides evidence for the interpenetration of the OIHP and the FI–SnO_2_ ETL. To further prove this, we performed cross-sectional transmission electron microscope (TEM) characterization (Supplementary Fig. 6) and energy-dispersive X-ray spectroscopy (EDX) analysis (Fig. 2e, f) of the two OIHP/ETL films. The sample specimens were carefully prepared using focus ion beam (FIB) nanofabrication. Consistent with the cross-sectional SEM images, the thickness of the FI–SnO_2_ film (~95 nm) is approximately double that of the pristine SnO_2_ (~45 nm). Pb element, standing for OIHP, is evenly distributed in FI–SnO_2_, clearly revealing the interpenetration of SnO_2_ and OIHP. For comparison, in the OIHP/pristine SnO_2_ structure, a relatively sharp transition in element distribution is shown between the ETL and OIHP, indicative of normal contact between the two layers without notable interpenetration. Considering all these observations, it is reasonable to deduce that an interfacial reaction has occurred between the excess PbI_2_ in OIHP and the FA halide in FI–SnO_2_, leading to the formation of the OIHP phase within the FI–SnO_2_ ETL.

### Electronic structure of the interpenetrating SnO_2_–OIHP interface

**Fig. 3:**
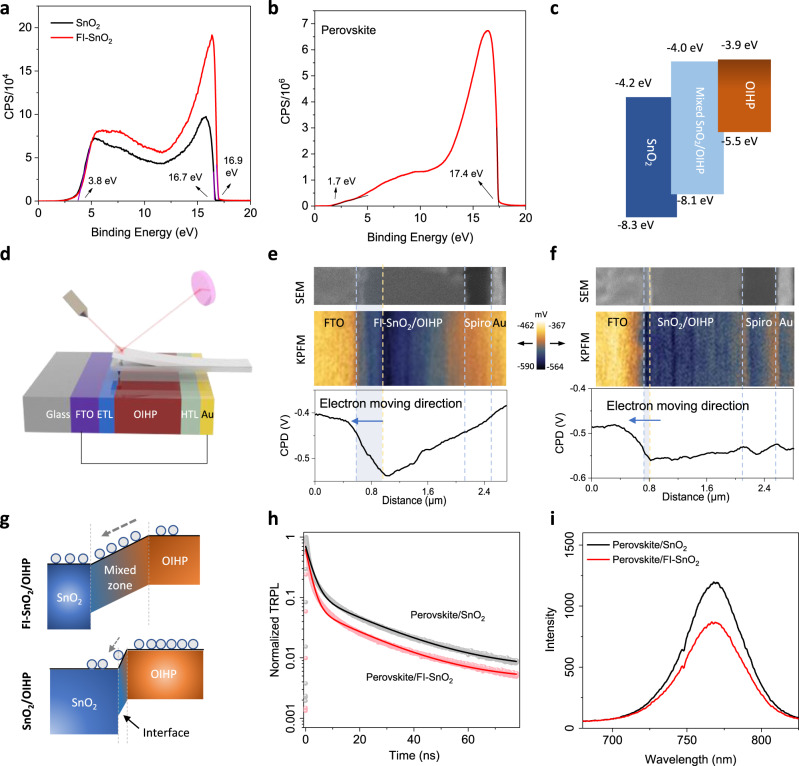
Electronic properties of SnO_2_–OIHP interfaces. UPS spectra showing the Fermi edge (left) and cutoff energy (right). **a** FI–SnO_2_ and pristine SnO_2_ ETLs and **b** OIHP. **c** Energy-level diagram across the OIHP/FI-ETL interface. **d** Schematic illustration of cross-sectional KPFM measurement under short-circuit condition. From top to bottom, SEM image, KPFM image, and contact potential difference (CPD; averaged) profile of the device on, **e** OIHP/FI–SnO_2_ interface and, **f** pristine OIHP/SnO_2_ interface. **g** Schematic illustration of electron transport characteristics near the OIHP/FI–SnO_2_ and pristine OIHP/SnO_2_ interfaces, respectively. **h** TRPL and **i** steady-state PL spectra of OIHP thin films on FI–SnO_2_ and pristine SnO_2_ ETLs.

Based on the above characterization results, we confirm that the OIHP/FI–SnO_2_ interface comprises a mixed interlayer of SnO_2_ and OIHP phases, as illustrated in Fig. 2a. Ultraviolet photoemission spectroscopy (UPS) was used to determine the energy levels of those layers. As shown in Fig. 3a, the surface of the FI–SnO_2_ ETL and the pristine SnO_2_ ETL have similar Fermi levels of 3.8 eV below the vacuum level, but the cutoff energy for FI–SnO_2_ ETL (16.9 eV) is larger than that for pristine SnO_2_ ETL (16.7 eV). Considering the photon energy (21.2 eV) of He–I_α_ radiation for the UPS measurements and the bandgap (4.1 eV) of SnO_2_, the VBM levels are −8.1 eV and −8.3 eV and the CBM levels are −4.2 eV and −4.0 eV, respectively, for FI–SnO_2_ and pristine SnO_2_. Based on these results, the energy-level diagram for the OIHP/FI–SnO_2_ interface is presented in Fig. 3c, which interestingly reveals a cascade electronic structure that is favorable for photocarriers transfer.

Furthermore, we employed in operando Kelvin probe force microscopy (KPFM) (Fig. 3d) to examine the potential profile along the cross-section in the device at the short-circuit condition under light illumination. KPFM has been proven to be a useful method for probe interface energetics in PSC devices^31^. Figure 3e, f is KPFM images of the FI–SnO_2_ and pristine SnO_2_ ETL-based devices, respectively, together with corresponding cross-sectional SEM images and contact potential difference (CPD) profiles. The related topological and phase atomic force microscopy (AFM) images are shown in Supplementary Fig. 7, demonstrating CPD results are independent of the surface topography^32,33^. The slope of the CPD profile suggests the existence of a p–n junction. The slope degree and range represent the driving force for exciton separation and the width of the depletion region, respectively. Compared with the small and narrow CPD drop at the OIHP/pristine SnO_2_ interface, the drop magnitude at the OIHP/FI–SnO_2_ interface is much larger (0.1 V vs 0.03 V), and the depletion region has penetrated into the OIHP layer. Based on the above results, we can deduce the photogenerated carrier transport behavior near the OIHP/ETL interface: as schematically illustrated in Fig. 3g, upon the device operation, the wider depletion region and larger potential difference near the OIHP/FI–SnO_2_ interface will greatly promote the separation of photogenerated carriers and the sequential collection of electrons. We then employed time-resolved photoluminescence (TRPL) and steady-state PL spectroscopy to study the carrier dynamics across the OIHP/ETL interface. The TRPL spectra in Fig. 3h show a faster biexponential PL decay for the OIHP/FI–SnO_2_ ETL, with lifetimes of *τ*_1_ = 1.8 ns and *τ*_2_ = 19.2 ns, as compared to *τ*_1_ = 2.3 ns and *τ*_2_ = 20.5 ns for the OIHP/pristine SnO_2_ ETL. The shorter photocarrier recombination times is consistent with the more efficient PL quenching in the OIHP/FI–SnO_2_ case, as revealed by the steady-state PL results in Fig. 3i. The enhanced charge dynamics of the OIHP/FI–SnO_2_ interface is attributed to its high structural and electronic integrity.

### Device performance of perovskite solar cells in rigid and flexible versions

**Fig. 4:**
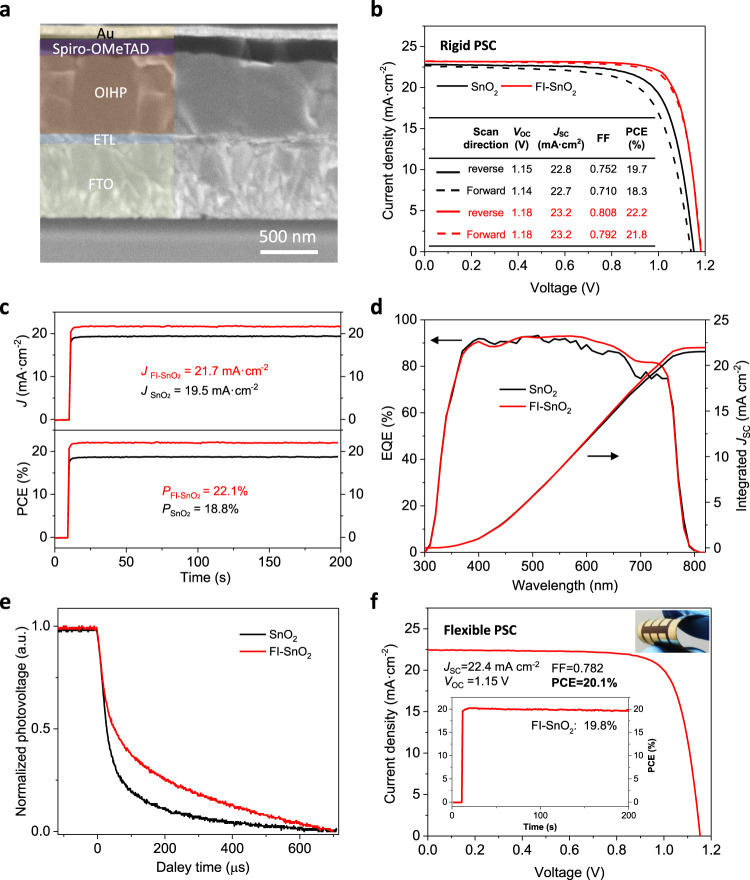
Photovoltaic performance of rigid and flexible solar cell devices. **a** Cross-sectional SEM image of a rigid PSC device based on FI–SnO_2_ ETL. **b** *J–V* curves (forward and reverse scans) of rigid PSCs based on FI–SnO_2_ and pristine SnO_2_ ETLs. The inset table shows the extracted PV parameters. **c** MPP current/PCE outputs, and **d** EQE spectra of the champion rigid PSCs based on FI–SnO_2_ and pristine SnO_2_ ETLs. **e** Transient photovoltage decays for PSCs based on FI–SnO_2_ and pristine SnO_2_ ETLs. **f** *J–V* curve and MPP PCE output of the champion flexible PSCs based on FI–SnO_2_ ETL. Inset is the photograph of a flexible PSC device.

Rigid PSCs (on FTO-coated glass substrates) were fabricated after depositions of spiro-OMeTAD hole transporting layers (HTLs) and Au contacts on the as-formed OIHP/ETL film structures. Figure 4a shows the cross-sectional SEM image of a typical PSC, where all the layers are labeled. The current density–voltage (*J*–*V*) curves (forward and reverse scans) of the champion PSCs based on FI–SnO_2_ and pristine SnO_2_ ETLs are compared in Fig. 4b. The FI–SnO_2_-based PSC shows a high reverse-scan PCE of 22.2% with an open-circuit voltage (*V*_OC_) of 1.18 V, a fill factor (FF) of 0.808 and a short-circuit current density (*J*_SC_) of 23.2 mA cm^−2^, all higher than those for the pristine–SnO_2_-based PSC (PCE: 19.7%; *V*_OC_:1.15 V; FF:0.752; *J*_SC_: 22.8 mA cm^−2^). The stabilized current/PCE outputs of the devices were further monitored at maximum-power-point (MPP) bias voltages, as shown in Fig. 4c. Stabilized PCEs of 22.1% and 18.8% are shown for PSCs based on the FI–SnO_2_ and pristine SnO_2_ ETLs, respectively. The external quantum efficiency (EQE) spectra for both devices are shown in Fig. 4d, where the integrated *J*_SC_ values are consistent with those extracted from the *J–V* curves.

The carrier dynamics in both devices was further characterized by monitoring transient photovoltage decays. As shown in Fig. 4e, the FI–SnO_2_-based device exhibits a carrier recombination lifetime *τ*_*r*_ (72 µs) that is much higher than that (30 µs) of the pristine–SnO_2_-based device. This is in good agreement with the enhanced integrity of the OIHP/FI–SnO_2_ interface and explains the higher device performance parameters. FI–SnO_2_ ETL is prepared at low temperatures, and therefore, it is amenable to deposition on flexible polymer substrates for the fabrication of flexible PSCs. Figure 4f and Supplementary Fig. 8 show the *J–V* curve (reverse scan) of the champion flexible PSC. Note that this device is made on a polyethylene naphthalate (PEN)/ITO substrate. A PCE of 20.1% is obtained under reverse *J–V* scan direction, with *J*_SC_ of 22.4 mA cm^−2^, *V*_OC_ of 1.15 V, and FF of 0.782. Under forward scan direction, the PCE is 19.6%, with *J*_SC_ of 22.4 mA cm^−2^, *V*_OC_ of 1.14 V, and FF of 0.769. Upon MPP monitoring, a stabilized PCE of 19.8% is achieved. All these PV parameters are at the state-of-the-art level for flexible PSCs^34,35^.

### Operational stability and mechanical endurance of perovskite solar cells

**Fig. 5:**
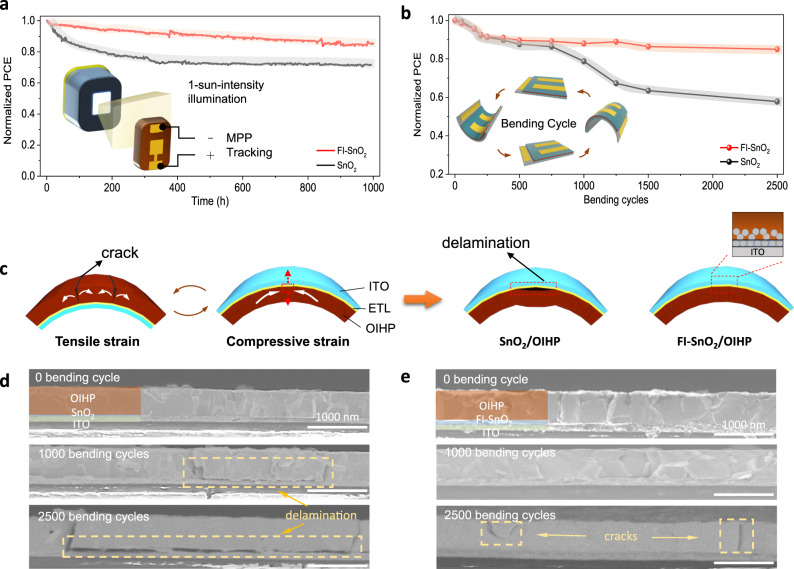
Operational stability and mechanical durability of solar cell devices. **a** Operational stability of rigid PSC devices based on FI–SnO_2_ and pristine SnO_2_ ETLs under continuous light illumination (one-sun intensity illumination; flowing N_2_; 40 °C). **b** Durability of flexible PSC devices based on FI–SnO_2_ and pristine SnO_2_ ETLs as a function of mechanical bending cycles (40% RH; ambient air; 25 °C; 3 mm minimum *r*); Inset illustrates a typical mechanical bending cycle. **c** Schematic illustrations showing the strains in OIHP layers under different bending states (left panel) and the film states after bending cycles (right panel). **d**, **e** Cross-section SEM images of flexible PEN/ITO/SnO_2_/OIHP film and PEN/ITO/FI–SnO_2_/OIHP film after different bending cycles.

The operational stability of rigid PSCs based on the FI–SnO_2_ and SnO_2_ ETLs is then compared. The initial PCEs are 21.1 and 19% for chosen PSCs based on FI–SnO_2_ and pristine SnO_2_ ETLs, respectively. These devices were tested under MPP tracking with one-sun-intensity illumination. As shown in Fig. 5a, the PSC based on FI–SnO_2_ ETL clearly exhibits much slower PCE decay, and after the 1000-h continuous operation, 82% of the initial PCE was retained, demonstrating the long-term operational stability. For comparison, the PSC based on pristine SnO_2_ ETL shows a typical rapid PCE degradation during the initial period of the test (72% retention after 400 h). Such degradation has been attributed typically to the OIHP/ETL interface delamination^36^. In this context, our new interface design enhances the structural integrity of the OIHP/ETL interface, which leads to more operationally stable devices.

For flexible PSCs, repeated cyclic-bending fatigue tests were performed to evaluate the mechanical endurance. As illustrated in the inset of Fig. 5b, the minimum bending radius (*r*) is 3 mm, which corresponds to a maximum stress (σ) of 371 MPa, calculated using the following relation^37^:

1 $$\sigma = \frac{{Eh}}{{2r}}$$

where *E* is the Young’s modulus of the OIHP thin film and *h* is the thickness of the substrate (125 µm, neglecting other nanoscale layers in the device). Since the *E* for the OIHP composition used here is not known, the *E* value of 17.8 GPa for MAPbI_3_ OIHP is used here, as the elastic properties of OIHPs are governed by the 3D framework of the lead-iodide octahedra^37,38^. In a full bending cycle, the OIHP thin film is subjected to the following stressing sequence: 0 MPa (flat) to +371 MPa (convex; tension) to 0 MPa (flat) to −371 MPa (concave; compression), and back to 0 MPa (flat). As shown in Fig. 5c, while in the concave state, the compressive strain will transform into a peel-off stress perpendicular to the OIHP/ETL interface. We resort to cross-sectional SEM images to determine the film and interface changes during bending cycles. As seen in Fig. 5d, e, after 1000 bending cycles, the OIHP film begins to peel off from the pristine SnO_2_ ETL, while the FI–SnO_2_ OIHP/ETL doesn’t show obvious morphological change. When the bending cycle reaches 2500, the pristine OIHP/SnO_2_ film shows a more obvious delamination phenomenon, and the OIHP/FI–SnO_2_ interface is still intact, except for some cracks appearing in the OIHP. Cracks on the OIHP caused by tensile stress will not so significantly hinder the longitudinal transport of photogenerated carriers as the delamination of OIHP from ETL substrate caused by compressive stress. Thus after 2500 such bending cycles, the PCE still retains 85% of the initial value for the FI–SnO_2_ ETL-based device, while only 60% is retained for the pristine–SnO_2_-ETL-based control device. This improved mechanical durability is attributed to the interpenetrating characteristics of the OHIP/ETL interface in the FI–SnO_2_ ETL-based device. In fact, such diffuse interfaces are known to be more damage tolerant compared to sharp interfaces between two materials^39^.

## Discussion

We have developed an interpenetrating OIHP/ETL interface with enhanced structural integrity compared with its regular counterpart. We further characterized the interpenetrating characteristics of this interface using a set of advanced characterizations, well correlating the microstructure with the high performance and elucidating the underlying mechanisms. Incorporating this new interface enables the fabrication of efficient PSC devices that can deliver both long-term operational stability and high mechanical-fatigue endurance. This interface can not only retard ionic/molecular species to diffuse into the device, but also reduce the propensity for interfacial fracture, responsible for the device reliability enhancement. Furthermore, as identified by KPFM, the interpenetrating OIHP/ETL structure allows for more effective photocarriers separation and transport across the interface, compared to an abrupt OIHP/ETL interface, thus increasing the device PCE. While the case of OIHP/SnO_2_ is demonstrated for proof-of-concept, this study points to a new interface-engineering strategy that can improve perovskite photovoltaics and electronics technologies for a variety of applications.

For future research, we are aware that there are more advanced characterization techniques currently being developed for understanding perovskites at micrometer to atomic scales, including synchrotron X-ray imaging, high-resolution scanning TEM, and tomographic AFM^40–42^. By using these techniques, we expect to reveal the detailed microstructure–property correlations in such interpenetrating interfaces. Also, it is worth gaining more insights into the interface formation mechanisms, which will open up new possibilities in the facile synthesis of more complex interfaces and precise tailoring of electronic properties and mechanical robustness for perovskite devices.

## Methods

### Raw chemicals

All the chemicals were used as received from commercial companies, including PbI_2_ and PbBr_2_ (>99%, TCI, Japan), CH_3_NH_3_Br (MABr, 99.8%, Xi’an Polymer Light Technology Corp., China), NH_2_CH=NH_2_I (FAI, 99.8%, Xi’an Polymer Light Technology Corp., China), CsI (99.999%, Alfa Aesar, UK), SnCl_2_·2H_2_O (98–103%, Alfa Aesar, UK), Spiro-OMeTAD (99.7%, Lumtec Co., Taiwan), isopropanol (99.8%, Sinopharm Chemical Reagent Co., Ltd, China). 4-tert-butylpyridine (TBP, 96%), Bis (trifluoromethane) sulfonamide lithium salt (99.95%), and solvents acetonitrile (99.9%), dimethylformamide (DMF, 99.8%), dimethylsulfoxide (DMSO, 99.9%), α, α, α-Trifluorotoluene (≥99%) and chlorobenzene (CB, 99.8%) were all purchased from Sigma Aldrich (USA). FTO-coated glass substrates (7 Ω sq^−1^) and PEN/ITO flexible substrates (15 Ω sq^−1^; 125-μm thick) were purchased from Yingkou OPVtech New Energy Co. Ltd. (China).

### Preparation of precursors and solutions

For the traditional ETL, SnO_2_ nanocrystal colloidal solution was prepared by dissolving SnCl_2_·2H_2_O in anhydrous isopropanol (0.1 M) in an open reflux apparatus and stirred at 85 °C for 3 h. After standing at 40 °C for 3 h, the sol was aged for over 24 h at room temperature. For the FI–SnO_2_ ETL, the FAI powder has been dissolved in the above SnO_2_ sol with a concentration of 2 mg mL^−1^, 10 mg mL^−1^, 30 mg mL^−1^, and 60 mg mL^−1^. To prepare the Cs_0.04_(FA_0.84_MA_0.16_)_0.96_Pb(I_0.84_Br_0.16_)_3_ perovskite precursor, the mixed powder containing CsI (13.0 mg), FAI (180.6 mg), MABr (22.4 mg), PbBr_2_ (73.4 mg), and PbI_2_ (531.3 mg) were added into 1 mL mixed solvent of DMF and DMSO (volume ratio is 4:1), and stirring at 60 °C for 30 min. Precursor solution of HTL was prepared by dissolving 72.3 mg spiro-MeOTAD, 28.8 μL 4-tert-butylpyridine, 17.5 μL lithium bis (trifluoromethylsulphonyl) imide acetonitrile solution (520 mg mL^−1^) into 1 mL chlorobenzene.

### Device fabrication

Glass/FTO or the PEN/ITO substrates were etched with zinc powder and 4 M HCl to obtain the electrode pattern and then washed with cleaning fluid, deionized water, ethanol, and isopropanol sequentially. Subsequently, SnO_2_ sols were spin-coated on the substrates at 2000 rpm for 30 s, and then heated at 80 °C for 2 h to remove the solvent. All ETLs were then UVO-post-treated for 10 min. Finally, the ETL substrates were transferred to the glove box (H_2_O < 0.01 ppm, O_2_ < 0.01 ppm). To prepare the perovskite films, 50 μL above perovskite precursor was spread on the SnO_2_-ETL substrates, followed by a two-stage spin-coating process (1000 rpm for 10 s and 6000 rpm for 30 s). During the second spin-coating stage, 250 μL of α, α, α-trifluorotoluene was dripped on the spinning substrate 15 s prior to the end of the program. The substrates were then annealed at 100 °C for 50 min. Subsequently, HTL was deposited on top of the perovskite layer by spin coating at 3000 rpm for 30 s. Finally, a 60-nm Au electrode with an active area of 0.16 cm^2^ was thermally evaporated on top of the HTL.

### Materials characterization

The microstructures of the thin films were observed using field-emission SEM instruments (JSM-7401F, JEOL, Japan; Merlin, Zeiss, Germany). To obtain the cross-sectional SEM images, the flexible samples were embrittled with liquid nitrogen in the glove box (H_2_O < 0.01 ppm, O_2_ < 0.01 ppm) and fractured with two tweezers. The AFM, C-AFM images for ETL surfaces were obtained using an atomic force microscope (Dimension Icon, BRUKER, USA) in the contact mode. A multi-75E-G probe (PF TUNA) was used for the C-AFM detection. To obtain the samples for cross-sectional KPFM measurements, the devices were cleaved near the active area of the solar cells using a diamond cutter and then fractured by tension stress to expose the cross-section. The KPFM images of PSCs were obtained using the same model Bruker AFM as C-AFM measurement, while a conductive Antimony (n) doped Si Rtespa-300 probe (BRUKER, USA) with the frequency of 300 KHz and spring constants of 40 N m^-1^ was used for the detection. A standard AC mode, at a scanning rate of 1 Hz, AC voltage of 0.5 V, and frequency of 73 kHz, was used for amplitude modulation (AM-KPFM) measurement. The cross-sectional STEM specimen were prepared using a Focused Ion Beam nanofabrication platform (FEI Nanolab 600i, Thermo Fisher, USA). To protect the surface from damage during FIB milling, the cross-sectional surface was in situ-coated with platinum using an FEI gas injection system. Then the STEM images and EDS mappings were obtained on an aberration-corrected electron microscope (ARM200F, JEOL, Japan) at an acceleration voltage of 200 kV and beam convergence angle of 27 mrad. The collection angles were set to 68–280 and 45 mrad for HAADF and BF imaging, respectively. The TEM images of SnO_2_ nanocrystallines (scraped from the substrates) were performed on 2100 F (JEOL) instrument using an acceleration voltage of 200 KV. Steady-state and time-resolved PL spectra were recorded using a spectrophotometer (Varian Cary Eclipse Fluorescence, Agilent, USA) operated at 395 nm excitation. FTIR was obtained in attenuated total reflection (ATR) mode using an infrared spectrometer (V70, Bruker, USA). XPS was performed using Kratos Analytical spectrometer (AXIS ULTRA HAS, Kratos Analytical, UK) and all XPS spectra were shifted to account for sample charging using inorganic carbon at 284.80 eV as a reference. UPS was performed using Kratos Analytical spectrometer (AXIS ULTRA DLD, Kratos Analytical, UK), and mono-chromatized He–I_α_ radiation at 21.2 eV was used. The ToF–SIMS measurements (Model TOF–SIMS 5, ION-TOF GmbH, Germany) were performed with the pulsed primary ions from a GICB (10 keV) liquid-metal ion gun for the sputtering and a Bi_3_^++^ pulsed primary ion beam for the analysis (60 keV). The noninterlaced mode was used, with 1 s of sputtering followed by 3 s of analysis. The analysis area was 100  × 100 μm, and the sputter rate was calibrated with the SiO_2_ substrate on each batch of samples.

### Solar cell performance testing

The current density–voltage (*J–V*) characteristics of PSCs were measured by a Source Meter (2400, Keithley, USA) at the scan speed of 100 mV s^−1^ under AM 1.5 G one-sun illumination (100 mW cm^−2^) generated by a solar simulator (Oriel Sol3A Class AAA, Newport, USA) in air. The intensity was calibrated using a VLSI standard incorporated PN 91150V-KG3 Si reference cell. The active device area of PSCs during the measurements is 0.096 cm^2^ defined by a metal mask with a 0.35-cm diameter circular hole. Steady-state current/PCE outputs were measured using 2400 Source Meter (Keithley, USA) at voltages determined from the MPPs of the reverse-scan J–V curves. The EQE spectra were obtained using a quantum efficiency measurement system (Oriel IQE 200B, Newport, USA).

### Solar cell stability testing

For long-term operational stability tests, unencapsulated PSCs were placed in a sealed cell holder with a transparent quartz cover. A continuous flow of N_2_ gas was passed through the holder to minimize the water and oxygen content in the atmosphere. The PSCs were biased at the maximum-power-point voltage using a potentiostat under continuous one-sun-intensity white-LED illumination at around 45 °C. For mechanical bending tests, the ben radius is 3 mm. Each bending cycle consists of the device geometry status change of flat→convex→flat→concave→flat (as schematically illustrated in the inset of Fig. 5b). *J–V* curves were measured under the ambient condition to monitor the device performance decay.

### Reporting summary

Further information on research design is available in the Nature Research Reporting Summary linked to this article.

## Supplementary information

Supplementary Information

Solar Cells Reporting Summary

## Data Availability

The data that support the findings of this study are available from the corresponding authors upon reasonable request.
