# Supplementary material for: Interpenetrating interfaces for efficient perovskite solar cells with high operational stability and mechanical robustness
Source: Nat Commun. 2021 Feb 12;12:973. doi: 10.1038/s41467-021-21292-3 (PMC7881119; doi:10.1038/s41467-021-21292-3)
Supplement: Supplementary file 1 — Supplementary Information [file 41467_2021_21292_MOESM1_ESM.pdf]

Supplementary information

Interpenetrating Interfaces for Efficient Perovskite Solar Cells with High Operational Stability and Mechanical Robustness

Qingshun Dong[1,2], Chao Zhu[3,4], Min Chen[2], Chen Jiang[1], Jingya Guo[1], Yulin Feng[1], Zhenghong Dai[2], Srinivas K. Yadavalli[2], Mingyu Hu[2], Xun Cao[4], Yuqian Li[5], Yizhong Huang[4], Zheng Liu[4], Yantao Shi[1,*], Liduo Wang[6], Nitin P. Padture[2,*], Yuanyuan Zhou[2,7,*]

[1] State Key Laboratory of Fine Chemicals, Department of Chemistry, School of Chemical Engineering, Dalian University of Technology, Dalian, 116024, China

[2] School of Engineering, Brown University, Providence, RI 02906, USA

[3] SEU-FEI Nano-Pico Center, Key Laboratory of MEMS of Ministry of Education, Collaborative Innovation Center for Micro/Nano Fabrication, Device and System, Southeast University, Nanjing, 210096, China

[4] School of Materials Science and Engineering, Nanyang Technological University, Singapore, 639798, Singapore

[5] Analysis and Test Center, Beijing University of Chemical Technology, 100029, China

[6] Department of Chemistry, Tsinghua University, Beijing, 100084, China

[7] Department of Physics, Hong Kong Baptist University, Kowloon, Hong Kong

Correspondence should be addressed to yyzhou@hkbu.edu.hk (Y.Z.), nitin_padture@brown.edu (N.P.P.), and shiyantao@dlut.edu.cn (Y.S.)

Keywords: halide perovskite, solar cells, interface, stability, efficiency

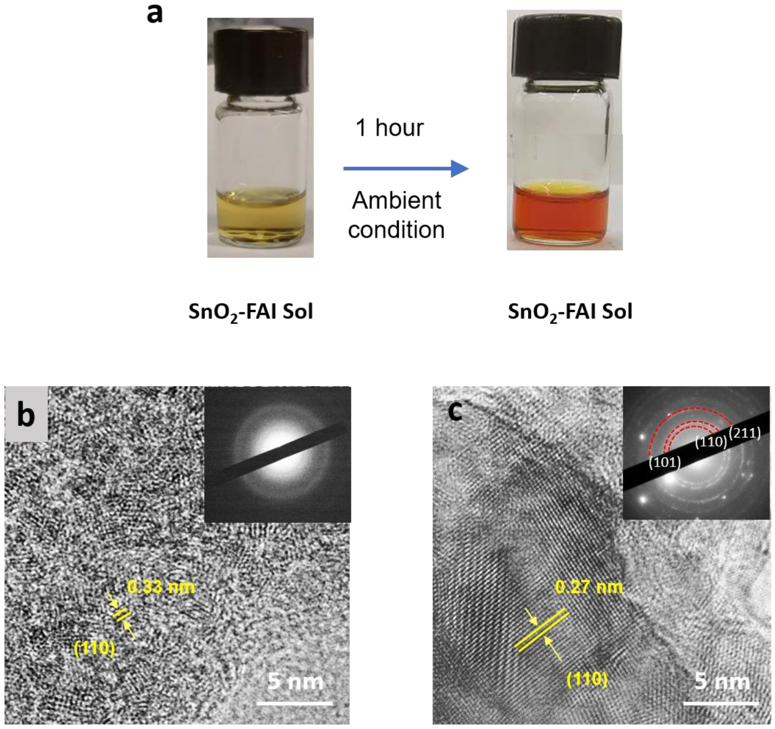

Supplementary Figure 1. **SnO_2 nanocrystals characteristics. a** Images of FI-SnO_2 (10 mg/mL) colloids: fresh (left) and stored under ambient condition for 1 h (right). HRTEM image of SnO_2 nanocrystallines scraped from **b** the pristine SnO_2 ETL, and **c** the FI-SnO_2 ETL. Insert: corresponding selected-area electron diffraction patterns.

The re-growth mechanism of the SnO_2 nanocrystals**.** When the FI-SnO_2 colloid placed under ambient condition for a longer time (~ 1 hour), the color changed from light brown to dark brown, indicating the formation of I_2, following the chemical reaction below:

$$O_2 + 4I^- + 2H_2O \rightarrow 4OH^- + 2I_2$$

the produced OH^- will promote further hydrolysis of -OR on the surface of SnO_2, triggering condensation and re-crystallization of SnO_2 nanocrystals (*Adv. Energy Mater*. 2019, 9, 1900834).

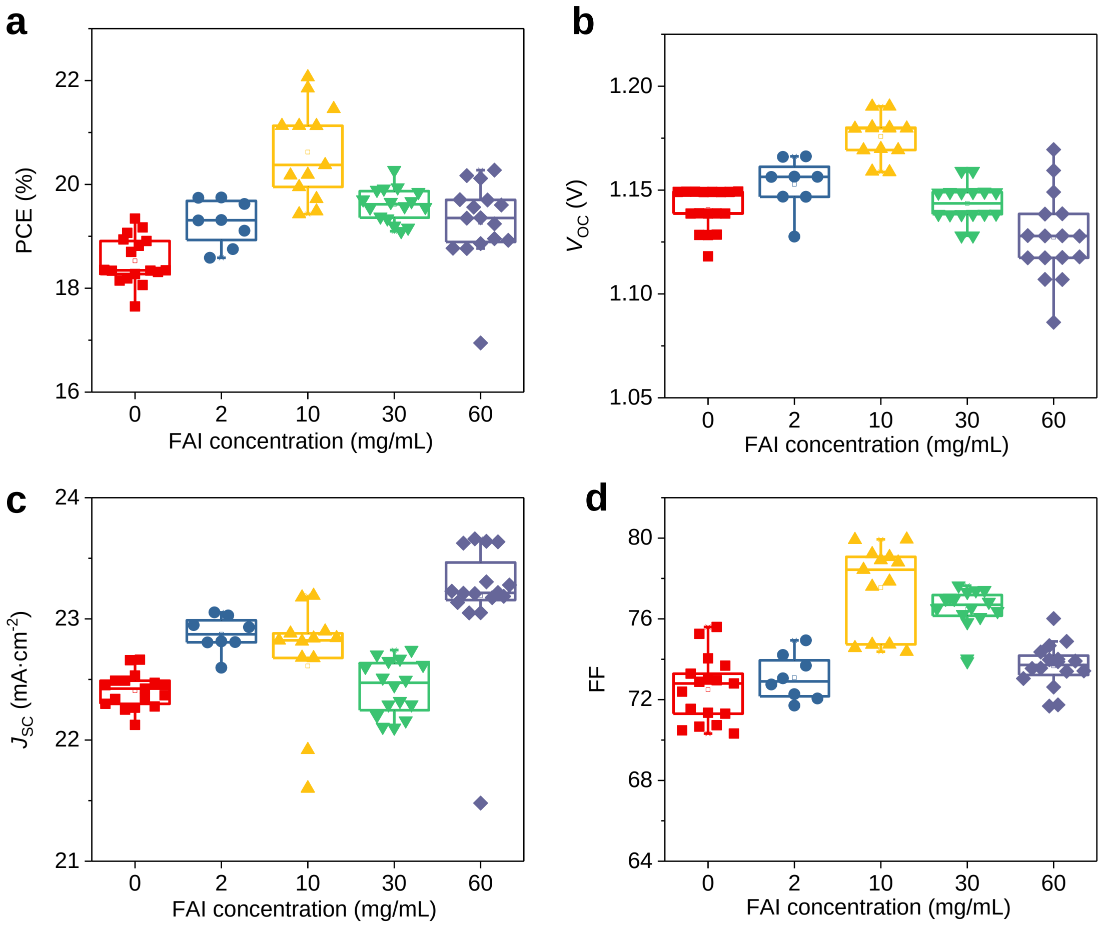

Supplementary Figure 2. **Optimization of FAI concentration in SnO_2 colloidal.** Statistics of the photovoltaic parameters: **a** PCE, **b** V_{OC}, **c** J_{SC}, and **d** FF of the devices on the ETLs from SnO_2 colloidal with different FAI concentrations.

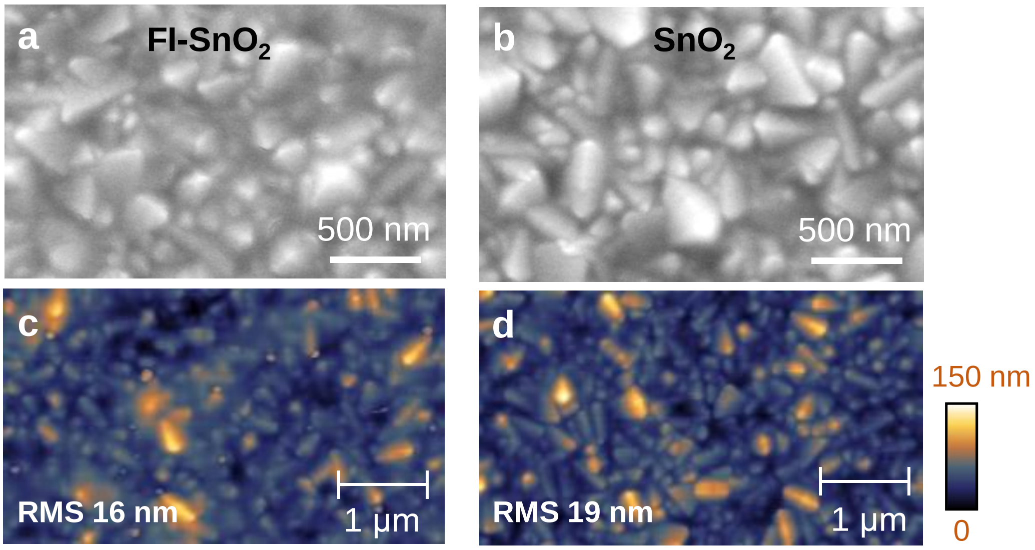

Supplementary Figure 3. **Surface morphology of ETLs. a, b** SEM images and **c, d** AFM mappings of the FI-SnO_2 (left) and SnO_2 (right) ETLs on FTO substrates.

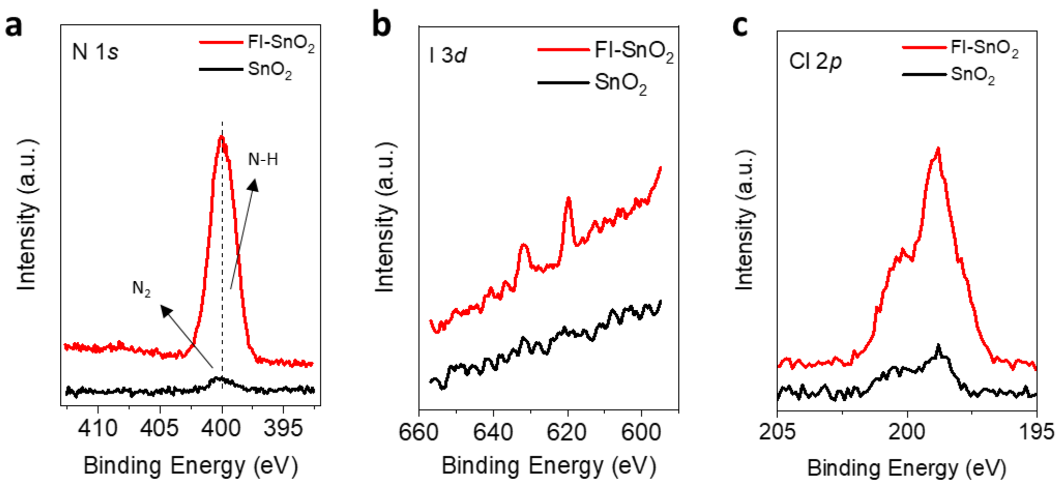

Supplementary Figure 4. **Composition analysis of ETLs.** XPS spectra of **a** N 1*s*, **b** I 3*d* and **c** Cl 2*p* for FI-SnO_2 and SnO_2 ETLs.

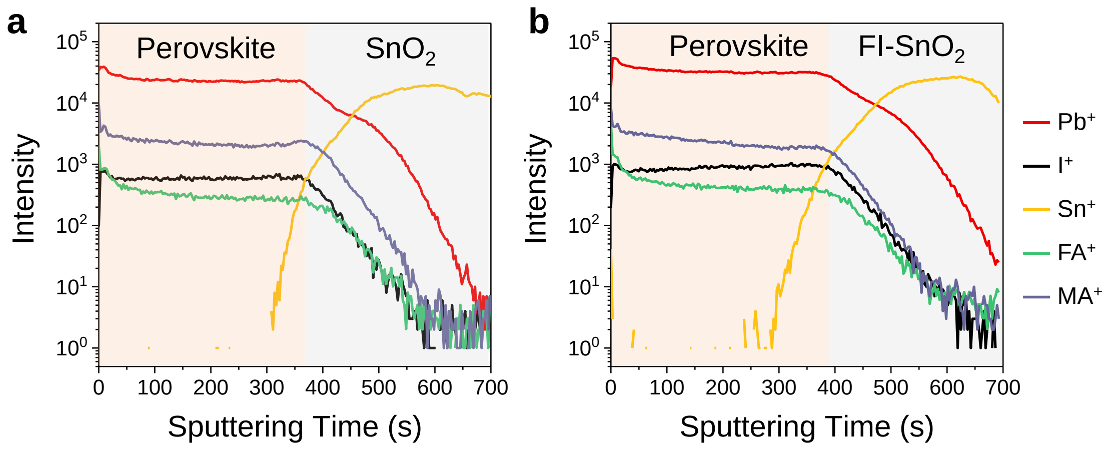

Supplementary Figure 5. **Structure characterization of OIHP/ETL interfaces.** The full ToF-SIMS elemental depth profiles: (a) OIHP/SnO_2 ETL; (b) OIHP/FI-SnO_2 ETL.

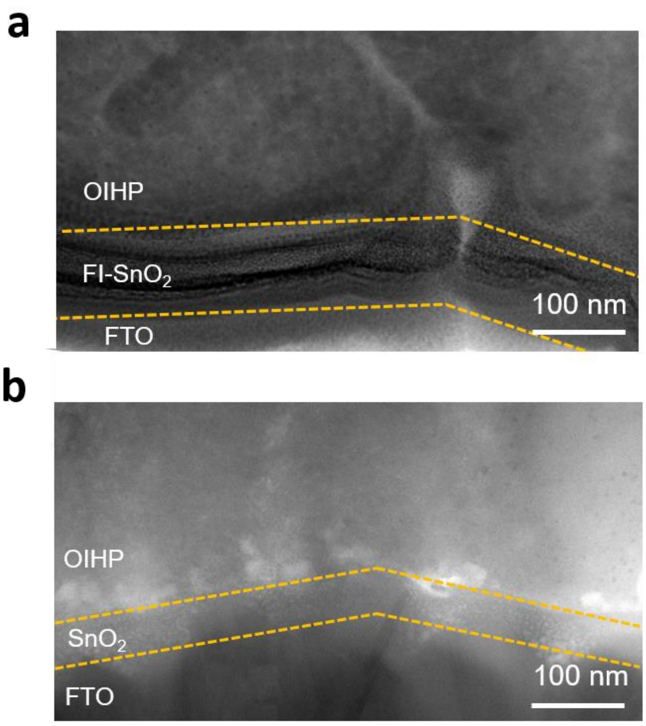

Supplementary Figure 6. **Microstructural ccharacterization of the OIHP/ETL interface.** Cross-sectional STEM images of OIHP films on, **a** the FI-SnO_2 ETL and **b** the pristine SnO_2 ETL.

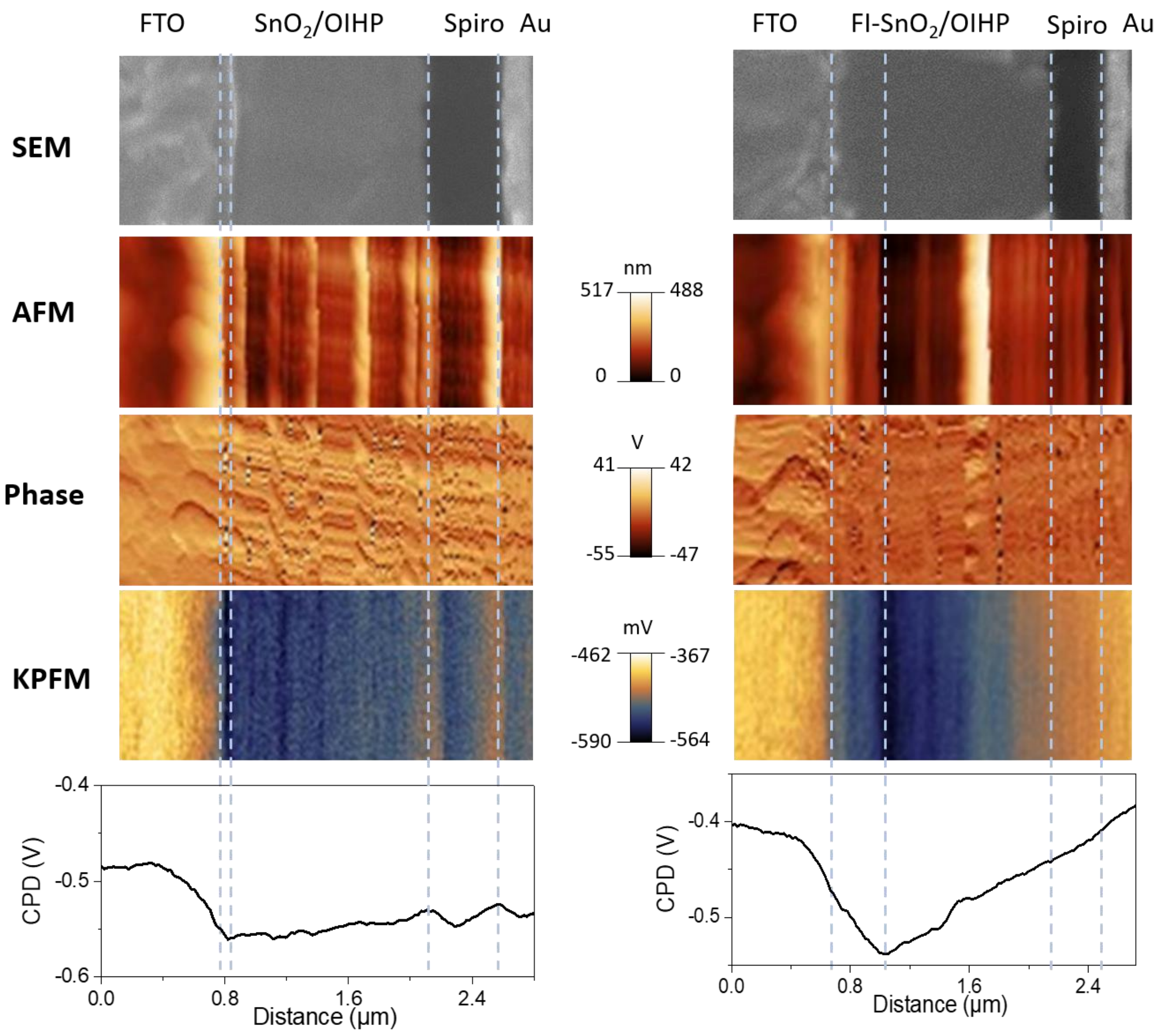

Supplementary Figure 7. **Nanoscale correlated characterization of morphology and eelectronic properties.** From top to bottom: SEM images, topographic AFM images, phase images, KPFM images and contact potential difference (CPD) profiles of the SnO_2/OIHP (left column) and the FI-SnO_2 OIHP (right column) devices.

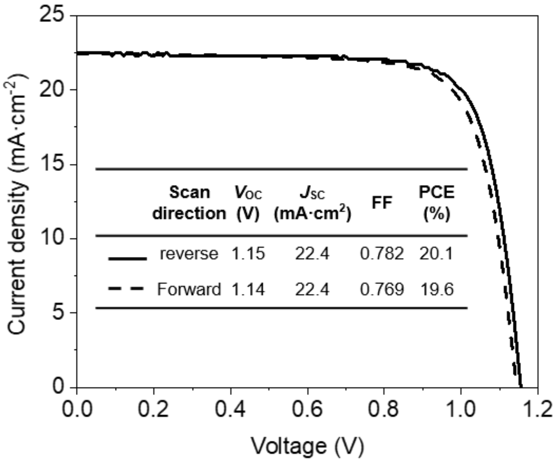

Supplementary Figure 8. **Photocurrent Hysteresis characteristics.** *J*-*V* curves (forward and reverse scans) of the champion flexible PSCs based on the FI-SnO_2 ETL.
