# Supplementary material for: Interpenetrating interfaces for efficient perovskite solar cells with high operational stability and mechanical robustness
Source: Nat Commun. 2021 Feb 12;12:973. doi: 10.1038/s41467-021-21292-3 (PMC7881119; doi:10.1038/s41467-021-21292-3)
Supplement: Supplementary file 2 — Solar Cells Reporting Summary [file 41467_2021_21292_MOESM2_ESM.pdf]

nature research

Corresponding author(s): Yuanyuan Zhou, Nitin P. Padture, Yantao Shi

Solar Cells Reporting Summary

Nature Research wishes to improve the reproducibility of the work that we publish. This form is intended for publication with all accepted papers reporting the characterization of photovoltaic devices and provides structure for consistency and transparency in reporting. Some list items might not apply to an individual manuscript, but all fields must be completed for clarity.

For further information on Nature Research policies, including our data availability policy, see Authors & Referees.

ü Experimental design

Please check: are the following details reported in the manuscript?

1. Dimensions

Item	Yes	No	Details
Area of the tested solar cells	☒	☐	0.16 cm2. See "Divice fabrication" in Methods section.
Method used to determine the device area	☒	☐	Defined by a non-reflective mask.

2. Current-voltage characterization

Item	Yes	No	Details
Current density-voltage (J-V) plots in both forward and backward direction	☒	☐	See Figure 4b.
Voltage scan conditions<br>*For instance: scan direction, speed, dwell times*	☒	☐	In "Solar cell performance testing" section.
Test environment<br>*For instance: characterization temperature, in air or in glove box*	☒	☐	In "Solar cell performance testing" section.
Protocol for preconditioning of the device before its characterization	☐	☒	No preconditioning, see "Solar cell performance testing" section.
Stability of the J-V characteristic<br>*Verified with time evolution of the maximum power point or with the photocurrent at maximum power point; see ref. 7 for details.*	☒	☐	See Figure 4c.

3. Hysteresis or any other unusual behaviour

Item	Yes	No	Details
Description of the unusual behaviour observed during the characterization	☒	☐	Negligible hysteresis. See Figure 4b.
Related experimental data	☒	☐	See Figure 4b.

4. Efficiency

Item	Yes	No	Details
External quantum efficiency (EQE) or incident photons to current efficiency (IPCE)	☒	☐	See Figure 4d.
A comparison between the integrated response under the standard reference spectrum and the response measure under the simulator	☒	☐	The integrated Jsc values are consistent with those from J-V measurements. see Figure 4c and 4d.
For tandem solar cells, the bias illumination and bias voltage used for each subcell	☐	☒	Not relevant to this work.

5. Calibration

Item	Yes	No	Details
Light source and reference cell or sensor used for the characterization	☒	☐	In "Solar cell performance testing" section.
Confirmation that the reference cell was calibrated and certified	☒	☐	Si-reference cell was certificated by NREL. See "Solar cell performance testing" section.

Calculation of spectral mismatch between the reference cell and the devices under test

- [ ] Yes
- [x] No

The Si-reference cell with KG3 window has a similar spectral corresponding range with that of PSC in our work, so we used mismatched factor of 1 for all devices.

6. Mask/aperture

Size of the mask/aperture used during testing

- [x] Yes
- [ ] No

0.096 cm2. See "Solar cell performance testing" section.

Variation of the measured short-circuit current density with the mask/aperture area

- [ ] Yes
- [x] No

The short-circuit current density doesn't vary with the mask/aperture area.

7. Performance certification

Identity of the independent certification laboratory that confirmed the photovoltaic performance

- [ ] Yes
- [x] No

This work mainy focuses on the mechanical reliability of PSCs.

A copy of any certificate(s)
Provide in Supplementary Information

- [ ] Yes
- [x] No

This work mainy focuses on the mechanical reliability of PSCs.

8. Statistics

Number of solar cells tested

- [x] Yes
- [ ] No

About 15 devices for each type of solar cell.

Statistical analysis of the device performance

- [x] Yes
- [ ] No

See supplementary Figure 2.

9. Long-term stability analysis

Type of analysis, bias conditions and environmental conditions
For instance: illumination type, temperature, atmosphere humidity, encapsulation method, preconditioning temperature

- [x] Yes
- [ ] No

See "Solar cell stability testing" section.
